# Toward Implantation‐Assisting Technologies: Lessons From In Vivo and Ex Vivo Models

**DOI:** 10.1002/rmb2.70042

**Published:** 2026-03-16

**Authors:** Takehiro Hiraoka, Yasushi Hirota, Masahito Ikawa

**Affiliations:** ^1^ Department of Obstetrics and Gynecology, Graduate School of Medicine The University of Tokyo Tokyo Japan; ^2^ Research Institute for Microbial Diseases Osaka University Osaka Japan; ^3^ Center for Advanced Modalities and DDS (CAMaD) Osaka University Osaka Japan; ^4^ Graduate School of Pharmaceutical Sciences Osaka University Osaka Japan; ^5^ The Institute of Medical Science The University of Tokyo Tokyo Japan; ^6^ Center for Infectious Disease Education and Research (CiDER) Osaka University Osaka Japan

## Abstract

**Background:**

Despite advances in assisted reproductive technology (ART), embryo implantation remains inefficient and represents a major barrier to successful pregnancy. Recurrent implantation failure persists even after transfer of high‐quality embryos, reflecting an incomplete understanding of the molecular mechanisms governing implantation.

**Methods:**

This review synthesizes current knowledge from genetically modified mouse models and an ex vivo system using authentic uterine tissue. Implantation is organized as a hierarchical, multistep process comprising acquisition of uterine receptivity, embryo attachment, and trophoblast invasion.

**Main Findings:**

Uterine receptivity is acquired through the action of progesterone signaling. Embryo attachment requires FOXA2‐mediated uterine gland maturation and activation of the LIF–STAT3 signaling axis. Subsequent invasion is driven by coordinated epithelial clearance, stromal differentiation, and embryonic activation. Disruption of these stage‐specific mechanisms leads to implantation failure. To overcome experimental limitations inherent to in vivo models, an ex vivo uterine system has been developed that preserves native tissue architecture and enables direct manipulation of embryo–uterine interactions.

**Conclusion:**

Conceptualizing implantation as a hierarchical process reveals discrete regulatory checkpoints and identifies implantation as a biologically tractable target. Integration of mechanistic insights with ex vivo platforms supports the development of implantation‐assisting technologies based on transient, trophectoderm‐targeted interventions in next‐generation reproductive medicine.

## 1 Background

Assisted reproductive technology (ART) has achieved consistently high fertilization rates, particularly with intracytoplasmic sperm injection (ICSI), which represents a major breakthrough in human reproductive medicine. In contrast, embryo implantation remains inefficient, with average success rates remaining below 40%–50% [1]. Recurrent implantation failure (RIF), defined by failure despite transfer of high‐quality embryos, therefore constitutes a persistent clinical challenge [2]. Its underlying pathophysiology is incompletely understood, and no definitive therapeutic strategy has been established. Advancing ART now critically depends on improved understanding and control of implantation.

A major obstacle to implantation research lies in the intrinsic biological properties of the process. Implantation occurs deep within the uterus and is difficult to observe directly in vivo. Moreover, unlike fertilization, which involves interactions between single cells, implantation is driven by coordinated multicellular interactions between the embryo and endometrium, consisting of diverse cell types. These interactions unfold through tightly regulated, sequential events within a narrow temporal window. Together, these features have hindered causal analysis and integrative dissection of molecular mechanisms, leaving implantation a longstanding biological black box.

**FIGURE 1:**
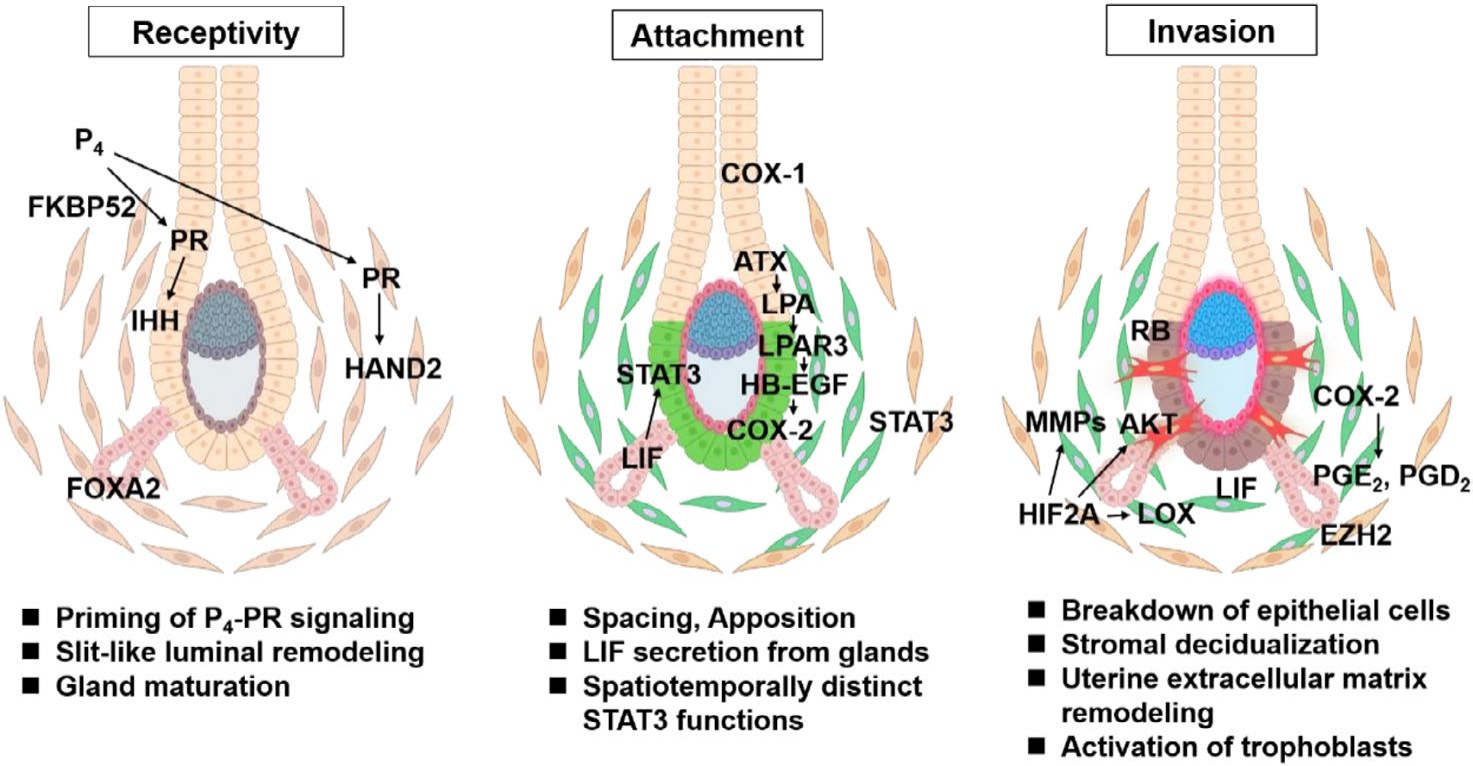
Spatiotemporal regulation of mouse embryo implantation. Implantation involves multiple steps, including acquisition of endometrial receptivity to the embryos, embryonic attachment, and trophoblast invasion. Several distinct pathways spatiotemporally coordinate these dynamic steps.

Importantly, implantation is not a single event but a multistep process [3, 4, 5, 6, 7, 8]. It proceeds through acquisition of uterine receptivity, embryo attachment to the luminal epithelium, and subsequent trophoblast invasion into the stromal compartment (Figure 1). Each phase is governed by distinct molecular programs and cellular dynamics. Nevertheless, implantation has often been approached clinically as a unified outcome, complicating identification of stage‐specific defects and obscuring rational therapeutic targeting.

Here, we conceptualize implantation as a hierarchical and interconnected molecular process encompassing receptivity, attachment, and invasion. We synthesize insights from previous mouse studies and introduce an ex vivo uterine system that enables direct interrogation of implantation dynamics. Together, these perspectives provide a framework for advancing mechanistic understanding of implantation and for developing strategies to address implantation failure.

## 2 Acquisition of Uterine Receptivity

Successful implantation requires acquisition of endometrial receptivity [4, 5], a transient state in which the endometrium becomes competent to accept the embryo. Receptivity occurs within a narrow temporal window and is tightly regulated by ovarian hormones, estrogen (E2) and progesterone (P4). E2 promotes epithelial proliferation, whereas P4 suppresses epithelial proliferation while stimulating stromal proliferation and then differentiation. Receptivity therefore requires a shift from estrogen to progesterone dominance; sustained estrogenic signaling prevents the receptive state. A defining feature of receptivity is the reversal of proliferative activity: epithelial proliferation ceases while stromal proliferation increases. We termed this reciprocal regulation proliferation–differentiation switching (PDS) [9]. PDS occurs during the implantation window in both mice and humans, indicating evolutionary conservation. Disruption of PDS causes implantation failure. In mouse models lacking PDS, epithelial proliferation persists and embryos fail to attach [10, 11, 12, 13, 14]. A proliferative epithelium thus functions as a barrier to attachment, establishing PDS as a functional requirement rather than a histological marker.

P4 signals through the progesterone receptor (PR) to coordinate epithelial and stromal responses [15]. Mouse studies have revealed that PR activation induces key downstream mediators including HAND2 [16], Indian hedgehog (IHH) [17, 18], and early growth response 1 (EGR1) [19], enabling precise epithelial–stromal crosstalk. This P4‐PR‐centered network synchronizes epithelial arrest and stromal activation to establish receptivity [20]. PR signaling is further modulated by epigenetic mechanisms. We have demonstrated that microRNA‐mediated regulation establishes uterine site‐specific PR responsiveness, indicating spatial as well as temporal control of receptivity [9]. Progesterone responsiveness is also regulated at the level of progesterone receptor (PR) complex stability. The immunophilin cochaperone FK506‐binding protein 52 (FKBP52) binds to HSP90 and PR, enhancing progesterone–PR signaling by stabilizing the mature receptor complex. FKBP52‐deficient female mice exhibit infertility specifically due to impaired uterine receptivity [21, 22, 23], despite normal ovulation and progesterone production. In these mice, attenuated PR signaling leads to defective proliferation–differentiation switching and persistent epithelial proliferation during the peri‐implantation period. Notably, this uterine progesterone resistance is reversible [23], as progesterone supplementation restores PR activity and rescues implantation, identifying FKBP52 as a key determinant of uterine progesterone sensitivity.

Clinically, progesterone‐dependent receptivity underlies current ART protocols [24, 25, 26]. Molecular understanding of PDS and PR signaling may enable refined endometrial assessment, optimized transfer timing, and identification of therapeutic targets.

## 3 Central Role of LIF–STAT3 Signaling in Mouse Embryo Attachment

Embryo attachment is a tightly regulated process controlled in both time and space [3, 4, 27]. Accumulating evidence indicates that attachment emerges through a hierarchical cascade involving uterine gland maturation, hormone‐triggered induction of secreted factors, and coordinated epithelial and stromal responses. Leukemia inhibitory factor (LIF) and its downstream effector STAT3 lie at the core of this cascade.

In mice, FOXA2‐dependent uterine gland maturation constitutes a critical prerequisite for embryo attachment [28, 29]. FOXA2 is selectively expressed in glandular epithelium and governs glandular branching and acquisition of secretory competence during the preimplantation period. During this stage, uterine glands undergo structural and functional remodeling to establish a permissive secretory environment. This process, termed the transitional phase, precedes nidatory E2 stimulation and represents a preparatory step toward implantation. Single‐cell analyses further reveal that periimplantation uterine glands are heterogeneous: robust LIF expression is confined to a distinct *Prss29*‐expressing subpopulation, indicating that attachment competence depends on glandular maturation state rather than gland presence per se [29]. Other studies have also been implicating the significance of gland maturation [30], including BMP signaling via the ACVR2A–SMAD1/SMAD5 axis, required for acquisition of receptivity [31, 32].

Within mature glands, nidatory E2 secretion from ovaries in mice induces LIF expression during the preimplantation phase [33]. LIF is a secreted cytokine of the IL‐6 family produced by uterine glands during the periimplantation period in mice [34]. In FOXA2‐deficient uteri, LIF induction fails despite intact E2 signaling, resulting in defective attachment [28, 29, 35]. These findings indicate that LIF induction is not driven by hormonal input alone but requires prior establishment of a mature glandular condition. FOXA2‐mediated gland maturation, therefore, licenses activation of the LIF–STAT3 pathway. Secreted LIF signals through the LIF receptor (LIFR) and the gp130 co‐receptor on the luminal epithelium [36, 37, 38, 39], activating the JAK–STAT pathway and driving STAT3 phosphorylation and nuclear translocation [40]. Genetic studies demonstrate that uterine‐specific deletion of *Stat3* completely abolishes embryo attachment, establishing STAT3 as an indispensable transcriptional regulator of this process. STAT3 has been shown to be indispensable for endometrial remodeling [41], whereas sustained STAT3 activation has been implicated in the development of adenomyosis [42], a chronic inflammatory gynecological disorder characterized by the presence of endometrium‐like tissue within the myometrium.

Importantly, STAT3 functions during embryo implantation are compartment‐specific yet mutually essential. Epithelial‐specific *Stat3* deletion disrupts luminal remodeling, including slit‐like lumen closure and implantation crypt formation, despite preserved proliferation–differentiation switching (PDS), thereby preventing attachment [43]. In contrast, stromal‐specific *Stat3* deletion permits epithelial morphological changes but leads to implantation failure due to dysregulated E2‐ER signaling and loss of PDS [43]. These observations demonstrate that successful attachment requires coordinated STAT3 activity in both epithelium and stroma.

LIF signaling also exhibits temporal complexity. Before attachment, gland‐derived LIF activates epithelial STAT3 to initiate attachment [44]. Following attachment, LIF expression is newly induced in the stroma at the embryo‐attachment site. Notably, exogenous LIF administration rescues implantation chamber formation and attachment in epithelial‐specific LIF‐deficient uteri but fails to sustain pregnancy in uterine‐specific LIF‐deficient uteri, despite improved attachment. This distinction suggests that epithelial LIF functions as an attachment trigger, whereas stromal LIF is required for post‐attachment progression and pregnancy maintenance [44].

Together, these findings from mouse studies establish embryo attachment as a multistep process requiring FOXA2‐dependent gland maturation, E2‐induced LIF expression, LIF–STAT3‐driven epithelial remodeling, and coordinated epithelial–stromal STAT3 signaling. Although the essential roles of these cascades in humans remain to be fully defined, this framework provides a mechanistic basis for understanding attachment failure.

## 4 Molecular Mechanisms Governing Embryo Invasion in Mice

Following embryo attachment, implantation progresses to the invasion phase, in which trophoblast cells breach the luminal epithelium and infiltrate the stroma, establishing the foundation for embryonic development and placentation. This transition relies on the local removal of the epithelial barrier at the attachment site, a prerequisite for trophoblast entry into the maternal compartment.

RB, encoded by the *Rb1* gene, is a critical regulator of epithelial clearance [45]. Although RB is classically recognized as a tumor suppressor that restrains cell cycle progression [46], it plays an essential role in epithelial elimination preceding invasion in the uterus. During normal implantation, epithelial cells undergo proliferative arrest accompanied by induction of TNF‐dependent necroptosis [47, 48], resulting in localized epithelial breakdown. RB not only enforces cell cycle arrest through repression of proliferation‐associated genes but also promotes epithelial elimination by activating TNF‐dependent cell death pathways. In *Rb1*‐deficient uteri, the epithelium persists as a physical barrier, preventing trophoblast access to the stroma and leading to developmental arrest due to invasion failure.

Prostaglandin synthesis mediated by cyclooxygenase (COX) enzymes represents another essential regulatory mechanism [49, 50, 51]. COX‐1 and COX‐2 serve distinct functions in the endometrium, with COX‐1 primarily regulating embryo positioning and spacing, whereas COX‐2 is indispensable for invasion. A previous study has identified an embryo‐induced uterine epithelial signaling axis involving autotaxin (ATX)–mediated lysophosphatidic acid (LPA) production, activation of an LPA receptor LPAR3, and HB‐EGF induction that culminates in epithelial COX‐2 induction at attachment sites. Through prostaglandin production, COX‐2 integrates these upstream signals to regulate ensuing stromal decidualization [52]. COX‐2 is also robustly induced in the primary decidual zone (PDZ) immediately after attachment, driving production of prostaglandins such as PGE_2_ and PGD_2_. These lipid mediators activate cAMP signaling through EP2/EP4 and DP1 receptors, coordinating epithelial remodeling with stromal proliferation and differentiation. Through this action, COX‐2 enables synchronized epithelial clearance and decidualization required for invasion [50, 53, 54, 55].

Hypoxia‐inducible factor 2α (HIF2α) functions as an integrative regulator of the microenvironment for trophoblastic invasion [56]. HIF2α promotes degradation of the basement membrane and extracellular matrix separating epithelium and stroma through MT2‐MMP–mediated proteolysis and LOX‐dependent modulation of matrix architecture. In parallel, HIF2α stimulates angiogenesis and stromal activation via vascular endothelial growth factor (VEGF) and adrenomedullin (ADM), while activating PI3K–AKT signaling in the embryo to support trophoblast survival and invasive capacity. Thus, HIF2α couples maternal tissue remodeling with embryonic signaling to facilitate invasion [56].

Epigenetic regulation within the stroma further ensures proper progression of invasion [57]. The polycomb repressive complex 2 (PRC2) catalytic subunit EZH2 suppresses gene expression through trimethylation of histone H3 lysine 27 (H3K27me3) [58] and governs the transition of stromal cells from proliferation to differentiation into decidual cells. In the absence of EZH2, loss of H3K27me3 leads to sustained expression of cell cycle genes, failure of stromal differentiation, and disruption of the decidual environment required to support invasion [57]. Importantly, PRC2–H3K27me3‐targeting genes were dysregulated in the human endometrium obtained from RIF patients [57].

Collectively, embryo invasion emerges from coordinated regulation of epithelial clearance, extracellular matrix remodeling, stromal differentiation, and embryonic activation. RB, COX‐2, HIF2α, and EZH2 operate at distinct yet interconnected regulatory layers, and loss of any single component is sufficient to halt invasion. This phase therefore represents a decisive checkpoint of the implantation cascade, integrating maternal and embryonic programs to ensure successful establishment of pregnancy.

## 5 Recapitulation of Implantation Using a Mouse Ex Vivo Uterine Culture System

While in vivo mouse models have revealed key molecular mechanisms underlying implantation, experimental systems enabling direct observation and causal testing have long been lacking. Implantation occurs deep within the uterus, making real‐time visualization and temporal intervention exceedingly difficult even in mice.

Although genetically modified models are powerful for establishing the necessity of individual factors, systemic deletion of key implantation regulators such as STAT3, gp130, and LIFR results in embryonic or perinatal lethality [59, 60, 61], precluding analysis of implantation phenotypes in adult females. Conditional knockout approaches [62, 63] circumvent this limitation but are labor‐intensive and still poorly suited for temporal or reversible manipulation. In this context, in vitro models, utilizing authentic embryos, stem cell‐derived models, and endometrial organoids, have been instrumental in enabling mechanistic studies under controlled conditions [64, 65, 66, 67, 68, 69, 70, 71, 72, 73, 74, 75, 76, 77]. For example, Fujimura et al. reported a mouse in vitro implantation model using endometrial organoids comprising both epithelial and stromal components [78]. In parallel, several human in vitro implantation systems have recently been established based on human endometrial organoids [76, 79, 80, 81, 82]. While these advances have significantly expanded experimental accessibility and mechanistic resolution, understanding implantation within its native spatial and multicellular context ultimately requires preservation of authentic tissue architecture. At the same time, fully reproducing the integrated interactions at the implantation site in vitro—including vasculature, immune cells, and glandular structures—represents an area of ongoing development. These considerations underscore the value of complementary experimental platforms that emphasize physiological fidelity alongside experimental accessibility.

**FIGURE 2:**
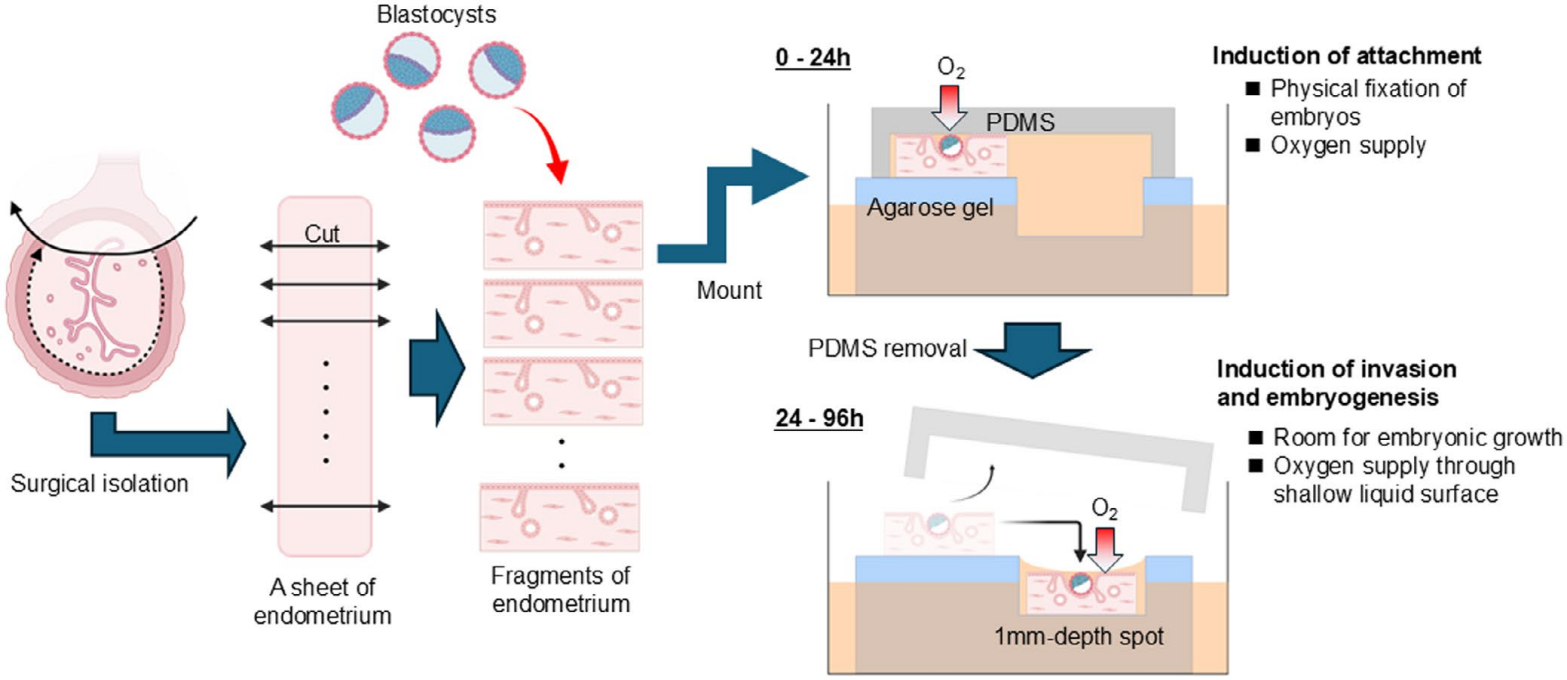
Establishment of an ex vivo uterine implantation system. Use of the authentic uterine tissues and embryos allowed for highly reproducible embryonic attachment and ensuing trophoblast invasion and embryogenesis. The key to implantation success was the oxygen supply from the embryonic/luminal end. PDMS, an oxygen‐permeable material, is necessary to physically settle the embryos on the endometrial fragments (0–24 h), and it is in turn removed after attachment to make room for subsequent embryonic growth under air‐liquid interface conditions through shallow medium surface (24–96 h).

To address these limitations, we established an ex vivo uterine culture system to preserve both the structural integrity and functional properties of intact uterine tissue [74] (Figure 2). Rather than reconstructing implantation in a simplified form, this system was designed to recapitulate in vivo phenomena as faithfully as possible. A central technical challenge was ensuring adequate oxygen and nutrient delivery. Inspired by air–liquid interface culture method used for ex vivo spermatogenesis [83, 84, 85], we adapted this strategy to maintain the periimplantation uterine environment. A custom culture device composed of polydimethylsiloxane (PDMS), an oxygen‐permeable material, was developed to permit efficient oxygen supply while facilitating physical contact between embryo and endometrium. Successful implantation required concurrent optimization of uterine tissue culture and embryonic culture, referring to recent advances in extended embryo culture beyond blastocyst stage in mice [66]. Extensive optimization included adjustment of ovarian hormone concentrations, comparison of FBS and KSR supplementation, regulation of tissue oxygen gradients through device orientation, and assessment of gravitational effects.

Under an optimized condition, the system recapitulates the full implantation sequence ex vivo, including embryo attachment, epithelial penetration, and trophoblast invasion [74]. Trophoblast differentiation, formation of trophoblast giant cell–like cells, and continuous post‐implantation embryonic development were also observed, enabling seamless modeling of implantation and early development while preserving uterine‐embryonic interactions.

The biological validity of the system was confirmed by comparison with in vivo implantation. Three‐dimensional histological analyses demonstrated preservation of PDS, vascular architecture, and the avascular region preceding vascular remodeling. Immune cell populations and glandular structures, both of which are essential for implantation [28, 29, 86, 87, 88, 89, 90], were also maintained. Single‐cell RNA sequencing further revealed close concordance in cellular composition and gene expression profiles with in vivo tissue, indicating that the system captures key features of a miniaturized physiological unit.

**FIGURE 3:**
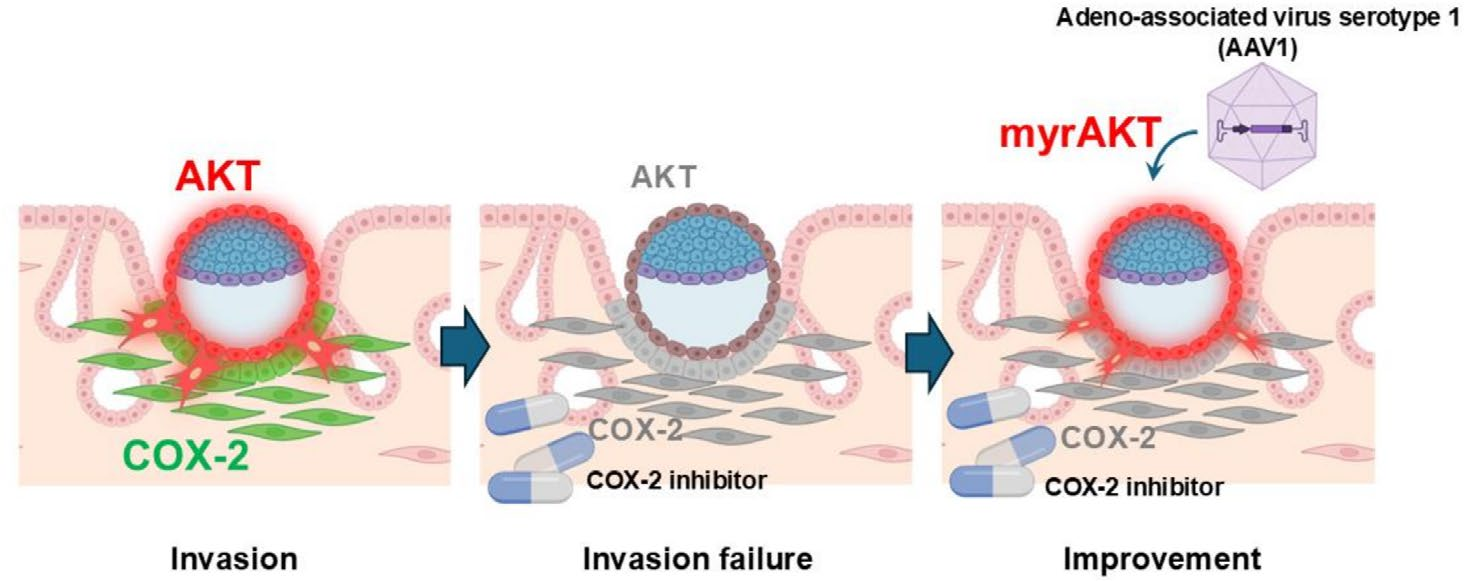
Ex vivo implantation analysis. A representative analytic model of ex vivo implantation screening is performed by the use of a COX‐2 inhibitor celecoxib and adeno‐associated virus 1 (AAV1). Trophectoderm‐specific introduction of a constitutively active mutant form of AKT1 (myrAKT1) ameliorated trophoblast invasion in a celecoxib‐induced defective implantation environment in the uterus.

A major advantage of this platform is the ability to facilitate assessment of maternal and embryonic compartments independently, allowing precise dissection of bidirectional signaling. One illustrative example is the interaction between maternal cyclooxygenase‐2 (COX‐2) and embryonic AKT signaling during invasion. In the ex vivo system, attachment site‐specific COX‐2 induction was faithfully recapitulated. Pharmacological inhibition of COX‐2 permitted embryo attachment but blocked subsequent trophoblast invasion, consistent with in vivo findings [50, 91]. Further analyses revealed that uterine COX‐2 activity activates embryonic AKT signaling [74], a pathway previously implicated in trophoblast invasion and placental development [92, 93, 94, 95, 96, 97]. Notably, trophectoderm (TE)/trophoblast‐specific delivery [98] of myristoylated Akt1 (myrAKT1), a constitutively active form of mutant AKT1, via adeno‐associated virus serotype 1 (AAV1) ameliorated invasion and decidualization under COX‐2 inhibition [74] (Figure 3). These findings demonstrate that the system not only reproduces implantation events but also enables direct testing of causal links between maternal signals and embryonic responses. Additional embryo–uterine dialogues [99], such as the HB‐EGF–ERBB2/3–VANGL2 axis [100], may similarly be interrogated using this platform.

**FIGURE 4:**
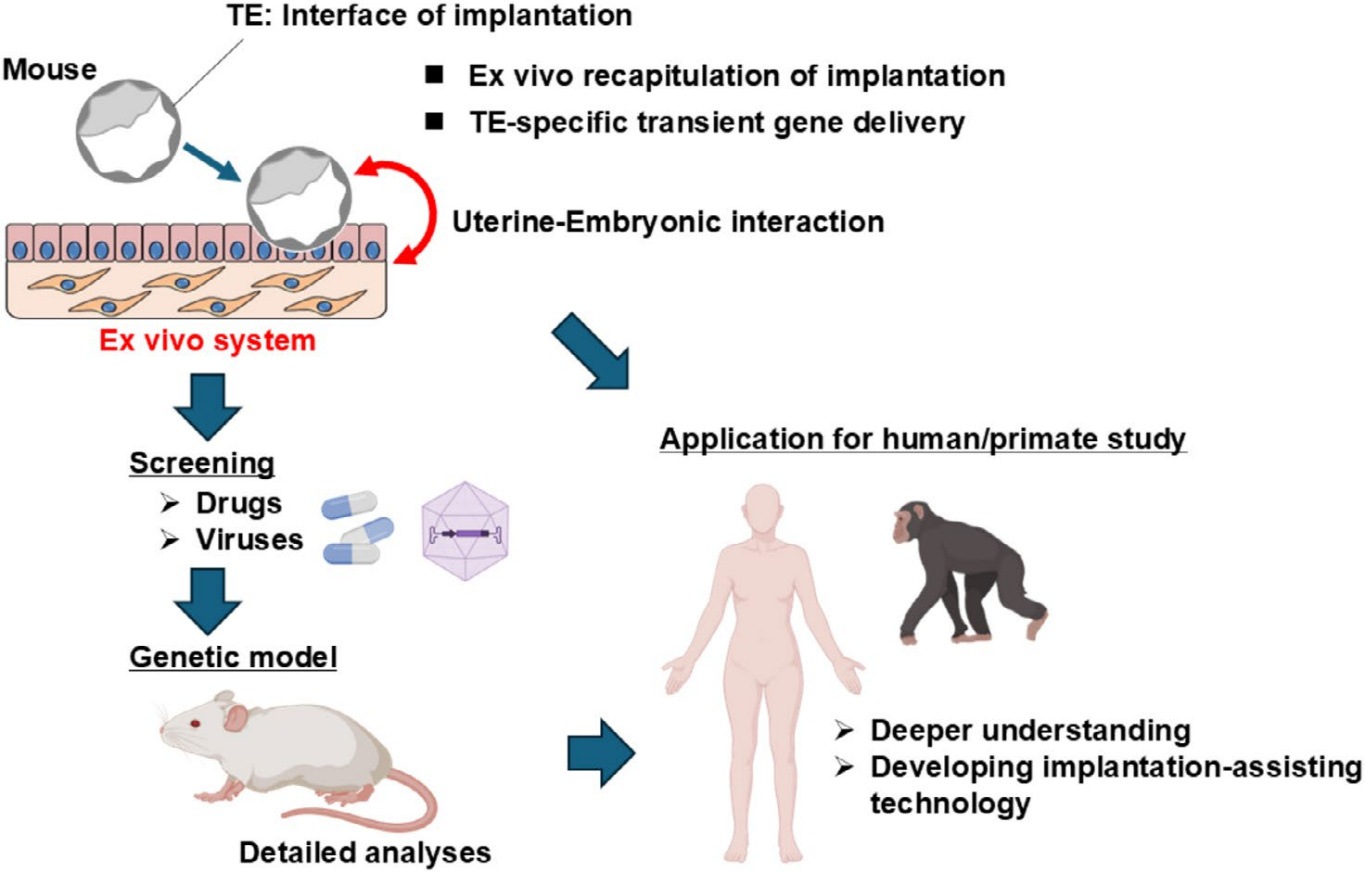
Scheme of study pipeline and clinical implications. Use of the current ex vivo system allows for preliminary implantation screening with drugs and viruses before establishing new gene‐knockout mouse lines. The system may also be applied for human and primate implantation study, which is intrinsically challenging.

Together, this ex vivo uterine implantation system provides a robust framework for integrative analysis of implantation mechanisms and offers a foundation for extension to clinically relevant models, including primates and humans (Figure 4). By enabling direct interrogation of embryo–uterine interactions, this approach has the potential to advance understanding of implantation failure and to inform the development of implantation‐assisting technologies.

## 6 Clinical Perspectives

A strategic approach to “implantation‐assisting technologies” in ART involves transient and reversible support during the limited temporal window of implantation, rather than permanent genetic modification. Because implantation is a phenomenon required only during early development, therapeutic intervention should ideally be localized and minimal. From this perspective, the concept of implantation “assistance” rather than genetic “therapy” captures an essential aspect of this technology.

TE serves as the executive tissue for attachment and invasion and is anatomically separated from the embryonic entity, making it a rational intervention target (Figure 4). Combining TE‐specific gene delivery technologies with the ex vivo system enables assessment of implantation intervention through genetic modulation of the TE. TE‐specific transient molecular supplementation using AAV [98] or lipid nanoparticles (LNPs) [101] represents a clinically suitable approach from a safety perspective. For example, a study demonstrated that among several AAV serotypes, only AAV1 efficiently delivered transgenes to the TE without transducing the inner cell mass (ICM) [98], which gives rise to the body of the embryo. Also, a study using LNP showed successful TE‐specific gene transduction without impacting ICM or offspring [101]. These aspects are important because any clinical translation would require strict validation of ICM exclusion. In addition, AAV vectors offer advantages over lentiviral vectors in that AAV predominantly remains episomal and carries a substantially lower risk of insertional mutagenesis than lentiviruses, which integrate into the host genome [102, 103]. This property is particularly suitable for implantation‐related applications, where transient and localized modulation is sufficient and permanent genomic alteration is undesirable. Moreover, LNPs are fully synthetic and devoid of viral components, and they eliminate the risk of vector‐derived genomic contamination inherent to viral production systems. LNPs may therefore offer an additional safety margin over AAV‐based approaches, particularly for clinical applications requiring short‐term and reversible molecular modulation [104, 105, 106]. By integrating molecular insights gained through systematic use of the ex vivo system with advances in safe gene delivery technologies, implantation may be emerging as a biologically tractable target in next‐generation reproductive medicine.

## Conflicts of Interest

The authors declare no conflicts of interest.

## Data Availability

The authors have nothing to report.
